# Sainsc: A Computational Tool for Segmentation‐Free Analysis of In Situ Capture Data

**DOI:** 10.1002/smtd.202401123

**Published:** 2024-11-12

**Authors:** Niklas Müller‐Bötticher, Sebastian Tiesmeyer, Roland Eils, Naveed Ishaque

**Affiliations:** ^1^ Center of Digital Health Berlin Institute of Health at Charité – Universitätsmedizin Berlin Charitéplatz 1 10117 Berlin Germany; ^2^ Department of Mathematics and Computer Science Freie Universität Berlin Arnimallee 14 14195 Berlin Germany; ^3^ Health Data Science Unit Heidelberg University Hospital and BioQuant University of Heidelberg Im Neuenheimer Feld 267 69120 Heidelberg Germany

**Keywords:** bioinformatics, cell type annotation, in situ capture spatial transcriptomics, segmentation‐free, spatial biology, spatial omics

## Abstract

Spatially resolved transcriptomics (SRT) has become the method of choice for characterising the complexity of biomedical tissue samples. Until recently, scientists were restricted to SRT methods that can profile a limited set of target genes at high spatial resolution or transcriptome‐wide but at a low spatial resolution. Through recent developments, there are now methods that offer both subcellular spatial resolution and full transcriptome coverage. However, utilising these new methods' high spatial resolution and gene resolution remains elusive due to several factors, including low detection efficiency and high computational costs. Here, we present Sainsc (Segmentation‐free analysis of in situ capture data), which combines a cell‐segmentation‐free approach with efficient data processing of transcriptome‐wide nanometre‐resolution spatial data. Sainsc can generate cell‐type maps with accurate cell‐type assignment at the nanometre scale, together with corresponding maps of the assignment scores that facilitate interpretation of the local confidence of cell‐type assignment. We demonstrate its utility and accuracy for different tissues and technologies. Compared to other methods, Sainsc requires lower computational resources and has scalable performance, enabling interactive data exploration. Sainsc is compatible with common data analysis frameworks and is available as open‐source software in multiple programming languages.

## Graphical abstract

Spatially resolved transcriptomics methods can now profile the entire transcriptome, at full organism scale with nanometre resolution. Analysis frameworks that can efficiently and intuitively process the massive data generated by these methods are lacking. Here Sainsc is presented, a computational tool for cell segmentation‐free analysis of spatially resolved transcriptomics data. Sainsc is robust, scalable, and available as open‐source software.

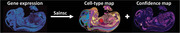

## 1 Introduction

Spatially resolved transcriptomics (SRT) is gaining popularity as a powerful tool for characterizing the complexity of biomedical tissue samples.^[^ ^1^ ^]^ This cutting‐edge technology allows researchers to map gene expression within the spatial context of tissue architecture, providing unprecedented insights into cellular heterogeneity and tissue organization.^[^ ^2^ ^]^ Unlike single‐cell RNA sequencing, spatial transcriptomics preserves spatial information, enabling the identification of cellular niches within tissues and organs. The spatial context is crucial for understanding complex biological processes such as development, disease progression, and response to therapy.^[^ ^3^ ^]^

SRT methods can be categorized broadly into two groups.^[^ ^2^, ^4^ ^]^ The first is imaging‐based methods that offer single‐molecule resolution through in situ hybridization (ISH)^[^ ^5^, ^6^ ^]^ or in situ sequencing (ISS)^[^ ^7^, ^8^ ^]^ for a selected number of target genes, normally in the range of 100–1000s. The second is in situ capture methods that incorporate spatial barcodes onto transcripts before sequencing, allowing whole transcriptome coverage but at limited spatial resolution^[^ ^9^, ^10^ ^]^ (e.g., 100 µm inter‐spot distance for Visium). The low spatial resolution of in situ capture methods complicates the spatial analysis of single cells, requiring deconvolution, imputation, and integration with external single‐cell transcriptomics resources.^[^ ^11^, ^12^, ^13^ ^]^ However, recent advancements in spatial transcriptomics have revolutionized the field by offering full transcriptome profiling at nanometre resolution through profiling methods such as Stereo‐seq, Seq‐Scope, Open‐ST, and Nova‐ST.^[^ ^14^, ^15^, ^16^, ^17^ ^]^ These high‐resolution techniques provide unique benefits, such as resolving transcriptome‐wide expression at subcellular levels, in some cases within the sub‐micron range.

However, these advancements also present several challenges. The sheer volume of data generated by these high‐resolution methods requires robust and scalable computational tools for efficient data processing and analysis. Furthermore, measured gene expression is sparse, potentially arising from a combination of low capture efficiency and a smaller total capture area compared with lower‐resolution methods.^[^ ^18^ ^]^ In the previous generation of supra‐cellular spatial resolution in situ capture SRTs, deconvolution of cell types was a common task. With the increased sub‐cellular spatial resolution of newer methods, there is the potential to assign gene expression at the single‐cell level.^[^ ^19^ ^]^ However, accurate cell segmentation remains elusive,^[^ ^20^ ^]^ making the correct assignment of measured transcripts to individual cells a nontrivial task. Addressing these computational challenges is essential for the widespread adoption and effective utilization of high‐resolution spatial transcriptomics in biomedical research.

To address the current computational challenges in processing and analyzing data from these methods, we developed Sainsc (pronounced “science,” segmentation‐free analysis of in situ capture data), which combines a cell segmentation‐free approach with efficient data processing of transcriptome‐wide nanometre resolution SRT data. Sainsc is tightly integrated with common data structures and frameworks for single‐cell and spatial data analysis in Python, rendering it highly accessible while outsourcing the computationally demanding tasks to the Rust programming language enabling efficient and scalable computational performance in analyzing full transcriptome nanometre‐scale data in native resolution. Furthermore, its segmentation‐free approach builds upon a Kernel Density Estimation (KDE) of gene expression, which reduces data sparsity and is suitable for classification tasks.^[^ ^21^ ^]^

We apply Sainsc to SRT data of several tissues, identifying cells missed by segmentation‐based analysis, show practical applications of spatial maps of the assignment score to explain the confidence of the model results, and demonstrate generalisability across profiling methods. Furthermore, Sainsc shows significant computational performance advantages over other competing tools, facilitating more efficient exploratory data analysis. Our implementation adheres to common data formats and analysis frameworks in the field to ensure interoperability, and the code is openly available on GitHub as Python and Julia packages.

## 2 Result

### 2.1 Sainsc—A Computation Tool for Segmentation‐Free Analysis of In Situ Capture Data

**Figure 1:**
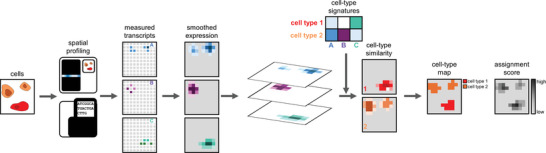
Sainsc workflow. Sainsc takes gene counts from spatially resolved transcriptomics platforms as input and models 2D gene expression using Kernel Density Estimation. Using reference cell‐type signatures (either from prior knowledge or calculated *de novo* from the data), it models cell‐type assignments using cosine similarity of the gene expression with the reference signatures. Each spot gets assigned a cell type and an assignment score is calculated to estimate the confidence of the assignment.

The Sainsc workflow was inspired by the SSAM and SSAM‐lite algorithms.^[^ ^19^, ^22^ ^]^ Briefly, SSAM models spatial gene expression as a density by applying the KDE, which can then be used for robust and accurate cell‐type assignment across the tissue. The computational steps can be broadly broken down into reading data, quality control, data preprocessing, modeling spatial gene expression, generating or acquiring cell‐type‐specific gene expression patterns, and assigning cell types to pixels to generate a cell‐type map (**Figure** **1**). Sainsc was developed to support efficient processing of in situ capture SRT data at native resolution while supporting imaging‐based SRT data by binning detected transcripts.

Sainsc's primary implementation is in Python and leans on Rust for optimized data structures, data processing, and safe multi‐threading support. The Rust elements enable computational efficiency, while the Python wrapper (using PyO3 for interfacing with Rust) enables accessibility to most computational life scientists. The main workflow is wrapped with a Python API and is provided with a broad range of convenience and plotting functions. Furthermore, we provide a Julia implementation of Sainsc's main functionality given its increasing popularity in the bioinformatics community.^[^ ^23^ ^]^ To ensure interoperability with the wider ecosystem of spatial and single‐cell analysis tools, Sainsc provides native support for reading established file formats (e.g., GEM files) and can output to community standard data structures (e.g., AnnData and SpatialData formats).^[^ ^24^, ^25^ ^]^

The Sainsc workflow comprises as follows; first, data are read into a custom Rust data structure (a hash map of sparse matrices of equal shape). The reading function supports popular file formats and creates a pixel structure representing the native resolution of the in situ capture SRT data. A sparse data format was chosen for memory‐efficient handling of sparse in situ capture SRT data. To support imaging‐based SRT or unsupported file formats, the Rust data structure can be constructed by providing a Python DataFrame. The optional next step involves filtering genes by count and visualizing gene expression over the tissue to select the region of interest for analysis. Then we model the gene expression density over the tissue by applying the KDE to all selected genes in Rust. Here, a kernel function (Sainsc has built‐in support for Gaussian and Epanechnikov) and the kernel bandwidth (in pixels or micrometers) are defined and the kernel is pre‐computed once, stored, and subsequently applied as required. To accelerate computation and prevent gene expression signal leakage, the Gaussian kernel is cropped at twice the bandwidth (by default). After the KDE is applied, the user can view the density of total gene expression over the tissue. The cell‐type map is generated in the final step. This step requires a cell‐type‐specific gene expression signature matrix, which can either be provided by the user (e.g., from existing single‐cell RNAseq data) or computed *de novo* from the data. *De novo* computation of cell‐type signatures from the SRT data follows a procedure similar to that of SSAM, where local maxima of gene expression are considered representative of cell gene expression profiles and clustered to identify cell‐type‐specific signatures.^[^ ^19^ ^]^ To minimize the memory and runtime requirements for the pixel‐level cell‐type assignment, only the subset of genes defined in the signature matrix are used, and small windows (by default, 500 × 500 with padding according to the kernel radius) are analyzed in parallel. Sainsc uses cosine similarity to identify the most similar cell‐type signature for cell‐type assignment to each element,^[^ ^26^ ^]^ which is then rendered as a colored pixel on the cell‐type map.

Cell‐type classification often entails variable class resolutions. For example, certain classes like neural endothelial cells are well‐defined with distinct transcriptional profiles. In contrast, other classes, such as various inhibitory or cortical‐pyramidal subtypes, require a much finer class resolution within the dataset. Therefore, the similarity between signatures may vary strongly. To effectively account for this, we have adopted a normalization strategy for the standard cosine similarity score. This adapted “assignment score” better reflects the local class resolution by scaling the difference in cosine similarity between the two best‐scoring cell types according to an upper bound based on the similarity between the two best signatures for each pixel.

To investigate the accuracy of spatial cell‐type assignment and assignment score we applied Sainsc to a synthetic dataset.^[^ ^27^ ^]^ (Figure S1, Supporting Information). The simulated data consisted of 100 replicates of 500 × 500 grids, containing 625 cells each assigned one of nine cell types of the mouse kidney. Spots were assigned gene expression based on the cell identity of the spot using a Poisson process with parameters inferred from single‐cell RNAseq data, and zero inflation was introduced uniformly at random, setting 20% of spots to zero counts. Sainsc demonstrated comparable accuracy to TopACT (both run at native resolution) and outperformed robust cell type decomposition (RCTD) with a bin size of 20 (Figure S1A,B, Supporting Information).^[^ ^28^ ^]^ Investigating the effect of filtering spots based on assignment scores revealed that the spots with the above median assignment scores obtained 0.995 accuracy for all but one cell type (Figure S1C). The spatial maps of the assignment score revealed distributions that visually corresponded to a realistic representation of cellular architecture given the ground truth setting (Figure S1D–G, Supporting Information).

### 2.2 Sainsc Reconstructs Accurate Cell‐Type Maps of Mouse Whole Embryo and Mouse Brain at Sub‐Micron Resolution

**Figure 2:**
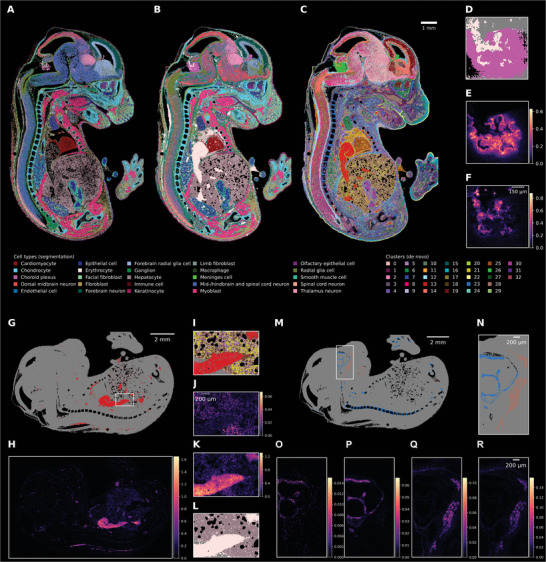
Sainsc enables whole organism (mouse embryo) cell type assignment at subcellular resolution. A) Segmentation‐based cell‐type assignment from Chen et al.^[^ ^14^ ^]^ B) a supervised cell‐type map using Sainsc with cell‐type signatures extracted from the segmentation‐based data and C) an unsupervised cell‐type map using Sainsc with de novo signatures. D–F) Choroid plexus region of the fourth ventricle for Sainsc supervised cell type assignment showing D) the structuring of the choroid plexus and its supply with erythrocytes highlighted by the expression of marker genes E) *Ttr* (choroid plexus) and F) *Hbb‐bs* (erythrocytes). G–L) Distribution of erythrocytes throughout the embryo G) in the unsupervised approach (cluster 13) validated by H) the erythrocyte marker gene *Hbb‐bs*. I–L) The region showing parts of the spleen and liver for I) the unsupervised approach, the expression of J) hepatocyte marker albumin (*Alb*), and K) erythrocyte marker *Hbb‐bs*, and L) erythrocytes and hepatocytes in the supervised cell‐type assignment M,N) Assignment of Cluster 23 and 28 correspond to chondrocytes and ossification activity, respectively as validated by the expression (KDE) of O) *Sox9*, P) *Col2a1*, Q) *Col1a1*, and R) *Col1a2*.

To investigate the usability of Sainsc with challenging real‐world data, we applied it to a section of an E16.5 whole mouse embryo data profiled using Stereo‐seq.^[^ ^14^ ^]^ The sample has 28633 genes detected in 10582619673 reads generated over a large field of view (≈1.4 × 0.9 cm), representing one of the largest in situ capture SRT datasets. We used Sainsc to perform both unsupervised and supervised analysis at native resolution and compared the output to previously published cell‐type maps (**Figure** **2**). The supervised analysis resulted in highly similar cell‐type maps (Figure 2A,B). We observed increased sensitivity across the section, including the choroid plexus cells at the fourth ventricle, which was further validated by its marker gene *Ttr* (Figure 2D,E). We believe that this increased sensitivity is a direct consequence of the cell segmentation‐free analysis of gene expression in Sainsc. Most notably, Sainsc was able to identify many more erythrocytes compared to the original study, with the highest accumulation of signal in expected organs: the spleen, liver, and umbilical cord. Interestingly, we found erythrocytes in the choroid plexus at the fourth ventricle (Figure 2D). We have previously demonstrated that current cell‐segmentation algorithms struggle with segmenting choroid plexus cells.^[^ ^19^ ^]^ The choroid plexus produces and secretes the majority of the cerebral spinal fluid and consists of ependymal cells surrounding a core of capillaries,^[^ ^29^ ^]^ rationalizing Sainsc's detection of erythrocytes adjacent to ependymal cells. The prediction of erythrocytes by Sainsc was supported by the expression of erythrocyte marker gene encoding for hemoglobin beta adult S chain (*Hbb‐bs*) (Figure 2F).

While using existing cell‐type‐specific signatures can be a convenient way to transfer cell‐type labels, inherent differences between profiling technologies may warrant the need for unsupervised analysis. Here, Sainsc employs an unsupervised clustering approach to generate cell‐type signatures from the tissue itself in the same manner as the SSAM algorithm.^[^ ^19^ ^]^ Briefly, under the assumption that positions of high gene expression are likely to be within cells, we sample these using local maxima detection. The local maxima are filtered based on their distance from each other to avoid sampling too many positions from the same cell. The gene expression profiles at these loci are then processed using standard single‐cell workflows and clustered using the Leiden algorithm. These clusters are representative of cell‐type‐specific gene expression patterns, and signatures are obtained by averaging the gene expression of each cluster. The unsupervised analysis of the mouse embryo section identified 33 clusters that corresponded to the expected organ structure (Figure 2C). The unsupervised analysis also resulted in superior detection of erythrocytes in the spleen and liver (Figure 2G–L). Erythrocytes are difficult to identify in a DAPI segmentation‐based analysis due to the lack of a nucleus in mature erythrocytes. We were also able to correctly distinguish ossifying regions (as demonstrated by expression of *Col1a1* and *Col1a2*)^[^ ^30^ ^]^ from other chondrocytes (expression of marker genes *Sox9* and *Col2a1*)^[^ ^31^ ^]^ (Figure 2M–R). These subclusters were previously reported by unsupervised analysis using FICTURE on the same dataset.^[^ ^32^ ^]^

**Figure 3:**
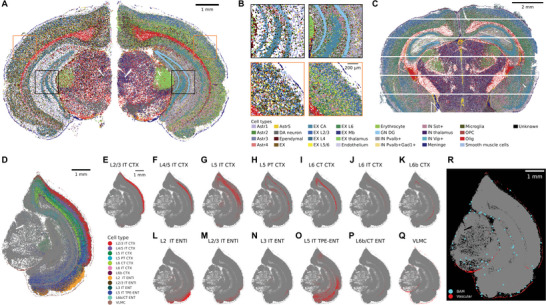
Sainsc accurately resolves cortical layering in Stereo‐seq data and identifies rare cell types in mouse brain sections. A,B) Comparison of segmentation‐based (left) and Sainsc (right) cell‐type maps of a coronal section of the mouse brain profiled by Stereo‐seq. C) Cell‐type signatures are transferrable to different technologies as demonstrated by cell‐type assignment in a coronal brain section profiled with Nova‐ST. D–Q) Using scRNAseq‐based signatures Sainsc resolves the intricate structures of the cortical plate as shown for E–P) the neuronal layers of the cortical plate and Q) the surrounding VLMC. R) Localization of identified border‐associated macrophages (cyan) is close to meningeal and vascular cells (red). Vascular subclasses include ABC NN, VLMC NN, Peri NN, Endo NN, and SMC NN. ABC, arachnoid barrier cells; CTX, isocortex; Endo, endothelial cells; ENT, entorhinal area, ENTl, lateral entorhinal area; IT, intratelencephalic; NN, non‐neuronal; Peri, pericytes; PT, pyramidal tract; SMC, smooth muscle cells; TPE, temporal association, perirhinal, and ectorhinal areas; VLMC, vascular leptomeningeal cells.

To further demonstrate the performance of Sainsc in delineating complex cell‐type organization, we applied Sainsc to mouse brain samples. First, we analyzed a Stereo‐seq dataset of a mouse brain coronal section. By performing supervised analysis using cell‐type signatures derived from the segmentation‐based analysis of the same tissue, we identified all major anatomical regions of the brain (**Figure** **3**A,B). Furthermore, we identified similar distributions of cell types in another mouse brain coronal section profiled by another full‐transcriptome sub‐micron resolution in situ capture SRT technology, Nova‐ST (Figure 3C).^[^ ^17^ ^]^ To further investigate the intricate cellular patterns, we generated a cell‐type map using signatures from Yao et al.,^[^ ^33^ ^]^ which contains cell‐type‐specific gene expression signatures for layer‐specific neurons in the isocortex and hippocampal formation regions of the cortical plate. The cell‐type map finely resolved the locations of numerous layer‐ and region‐specific neurons as well as the fine lining of vascular and leptomeningeal cells (VLMC) at the border of the tissue (Figure 3D–Q). This demonstrates that Sainsc can leverage cell‐type signatures obtained by different technologies, such as single‐cell RNAseq (scRNAseq) or other SRT technologies, to generate subcellular cell‐type classification.

Prior studies have highlighted the difficulty of finding rare cells such as macrophages in the central nervous system.^[^ ^27^ ^]^ To investigate the localization of border‐associated macrophages (BAMs), we annotated spots using the cell‐type signatures of Yao et al.^[^ ^34^ ^]^ The identified BAMs were distributed in the expected meningeal regions and perivascular space (Figure 3R).^[^ ^35^ ^]^ In particular, we identified BAMs localizing to the VLMC border layer that were not reported in a previous characterization of macrophage distribution in the same dataset.^[^ ^27^ ^]^ The observed localization of BAMs is consistent with coronal sections of the mouse brain profiled using MERFISH.^[^ ^36^ ^]^

### 2.3 Sainsc Effectively Analyses Imaging‐Based SRT Data

**Figure 4:**
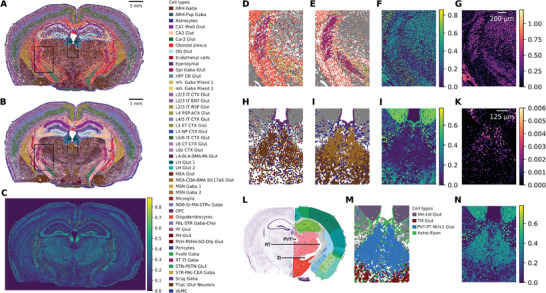
Sainsc is applicable to Xenium and the assignment score guides the identification of unrepresented cell types and regions. A) Reference cell‐type map based on segmentation, B) Sainsc's supervised cell‐type assignment, and C) assignment score of a Xenium coronal mouse brain section. D–G) Zoom in on the RT and ZI regions showing D) the original cell‐type assignments, E) Sainsc cell‐type assignments, F) Sainsc assignment score, and G) KDE of total gene expression. H–K) Misassigned cell type in the paraventricular nucleus of the thalamus (PVT), showing H) the original cell‐type assignments, I) Sainsc cell‐type assignments, J) Sainsc assignment score, and K) KDE of marker gene for PVT neurons, *Bcl11b*. L) Anatomical annotations of a similar section of the mouse brain from the Allen Mouse Brain Atlas and Allen Reference Atlas, mouse.brain‐map.org (https://atlas.brain‐map.org/atlas?atlas=1&plate=100960236, P56, Coronal section 72 of 132).^[^ ^38^, ^39^, ^40^, ^41^ ^]^

Imaging‐based SRT platforms with high sensitivity and larger gene panels are increasingly becoming commercially available. To demonstrate the applicability of Sainsc to these methods, we used it to analyze a publicly available Xenium dataset of a coronal section of the mouse brain (**Figure** **4**). We compared the cell‐type map produced by Sainsc to the cell types annotated in a previous study that used differentially expressed marker genes from scRNAseq data (Figure 4A,B).^[^ ^20^, ^34^, ^36^ ^]^ To ensure comparability, gene expression signatures were calculated from the segmented and annotated Xenium data and used for supervised cell‐type annotation with Sainsc. The resultant cell‐type maps showed remarkable similarity; however, we noted several differences. We observed inconsistent annotations in the anatomical region corresponding to the zona incerta (ZI) in the hypothalamus (Figure 4D,E,L). The ZI region contains GABAergic inhibitory neurons that have a shared developmental origin with GABAergic inhibitory neurons in the thalamic reticular nucleus (RT).^[^ ^34^, ^37^ ^]^ Salas et al.^[^ ^20^ ^]^ annotated neurons in the ZI as “inhibitory GABA mixed 2”, however, Sainsc more accurately annotated these as “RT ZI Gaba” with a high assignment score (Figure 4D).

Furthermore, we observed inconsistent annotations in the anatomical region corresponding to the paraventricular nucleus of the thalamus (PVT, Figure 4H–L). The PVT region contains glutamatergic neurons. Salas et al.^[^ ^20^ ^]^ annotated neurons in the PVT as “Thal Glut Neurons,” however, Sainsc annotated these as “MEA‐COA‐BMA Slc17a6 Glut.” The “Thal Glut Neurons” and “MEA‐COA‐BMA Slc17a6 Glut” gene expression signatures are highly similar (cosine similarity 0.79). In the Sainsc analysis, the assignment score of the PVT region was much lower than that of the surrounding thalamus (Figure 4J), which led us to suspect that the combination of the cell‐type signature matrix and Xenium gene panel was not optimal for annotating the PVT region. While we cannot change the gene panel, we investigated the effect of changing the cell‐type signature matrix. We chose to reannotate the sample using cell‐type signatures from the Yao et al. single‐cell RNA‐seq study, which included the signature of a PVT‐specific neuronal class, “PVT‐PT Ntrk1 Glut,” with the marker gene *Bcl11b*.^[^ ^34^ ^]^ Using this signature matrix, Sainsc correctly annotated the PVT region to contain the “PVT‐PT Ntrk1 Glut” neuronal class with a high assignment score (Figure 4M,N), which was validated by expression of the marker *Bcl11b* (Figure 4K).

We compared the performance of Sainsc to SSAM, which was developed for the segmentation‐free analysis of imaging‐based SRT data. The Xenium whole mouse brain slice dataset was reanalyzed with equivalent parameters for both methods using 1 µm sized‐bins and a bandwidth of 2.5 µm, as recommended for SSAM. The cell‐type assignments were highly similar with an adjusted Rand index of 0.86 and a normalized mutual information of 0.86 between SSAM (with “fast KDE”) and Sainsc (Figure S2A, Supporting Information). Furthermore, Sainsc demonstrated much better scalability than SSAM. SSAM with the “fast KDE” option finished the analysis in 23 min using up to 561 GB of RAM. Using the default KDE calculation in SSAM did not terminate the analysis within 7 days. Sainsc was able to perform the same analysis in less than 3 min (9× faster) using only 4.2 GB RAM (135× less) (Figure S2B–D, Supporting Information).

### 2.4 Computational Performance

**Figure 5:**
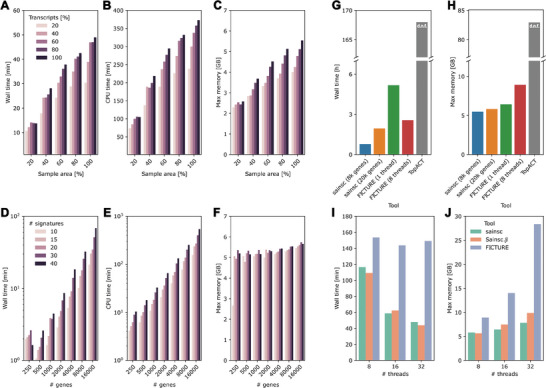
Sainsc is scalable and has low computational resource requirements. A) Wall time, B) CPU time, and C) maximum memory usage when analyzing Stereo‐seq coronal hemibrain data with downsampled tissue area and number of transcripts, and D) wall time, E) CPU time, and F) maximum memory usage for varying number of genes and cell types. Comparison of G) runtime and H) maximum memory usage of Sainsc, TopACT, and FICTURE. Comparison of I) runtime and J) maximum memory usage for Sainsc (Python and Julia implementation) and FICTURE with an increasing number of threads used for analysis. d.n.f.; did not finish.

A major problem associated with the newest generation of in situ capture SRT methods is handling and processing the large volumes of data generated. The combination of high spatial resolution and full transcriptome coverage increases processing time and memory requirements. Efficient data processing enables exploratory data analysis, e.g. by interactively modifying parameters to improve the output. We measured Sainsc's runtime and memory usage for varying dataset sizes by subsetting the tissue area and the number of detected transcripts, as well as varying numbers of genes and cell‐type signatures when analysing the Stereo‐seq mouse hemibrain section with 8 threads. Sainsc demonstrated scalable performance for wall time and CPU time concerning all parameters (**Figure** **5**A,B,D,E). While the memory usage also depends on these parameters, its increase with the number of genes and cell‐type signatures is more moderate than with the number of transcripts and the sample area (Figure 5C,F), demonstrating that memory usage is mostly dependent on the size of the dataset but not the analytical parameters. Often, only a few thousand highly/spatially variable genes are used for clustering and cell‐type assignment. Sainsc's wall time for a typical run (e.g., 2000 genes and 30 cell types) is less than 10 minutes, including data loading, therefore it effectively enables interactive data exploration.

Two recent computational tools, FICTURE and TopACT,^[^ ^27^, ^32^ ^]^ have also demonstrated accurate performance for creating cell‐type maps for SRT data. FICTURE claims to be efficient, while TopACT does not report processing time or memory requirements. We compared the computational performance of Sainsc with that of FICTURE and TopACT applied to the Stereo‐seq mouse brain sample (Figure 5G,H). To ensure comparability between FICTURE and Sainsc, we ran FICTURE using one core and 8 cores (as used by Sainsc) and Sainsc with 7972 genes and 19708 genes (as used by FICTURE). We recorded wall time and maximum memory usage in processing the Stereo‐seq adult mouse coronal hemibrain sample (Figure 5G,H). For all metrics, Sainsc performed similar to FICTURE and was substantially better than TopACT. In multi‐threaded mode, the wall time of Sainsc was less than 2 hours while FICTURE took ≈2.6 hours. TopACT running with 8 processes did not finish the analysis in the allotted time and the job was cancelled after 7 days. Furthermore, Sainsc required similar maximum memory usage to FICTURE in single‐threaded mode. The reduced runtime of FICTURE in multi‐threaded mode compared to its single‐threaded equivalent comes at a cost of increased memory usage however, ≈50% more than Sainsc. TopACT used substantially more memory (≈83 GB of RAM) during its runtime than either Sainsc or FICTURE. Additionally, Sainsc can further reduce the wall time by increasing the number of threads used for the analysis with only a small increase in memory usage, while for FICTURE the decreased runtime is minor compared to the increased memory consumption (Figure 5I,J).

Julia has been described as having the interactivity and syntax of scripting languages with the speed of compiled languages.^[^ ^42^ ^]^ Therefore, we also implemented the core functionality of Sainsc into a Julia package, Sainsc.jl. To demonstrate the correspondence between the Julia and the Python/Rust implementation we analyzed the Stereo‐seq mouse coronal hemibrain section with signatures obtained from segmented data and achieved near identical results (Figure S3, Supporting Information, discordantly classified pixels: 9.2 × 10^−5^%; median absolute difference of assigned pixels for the assignment score: 7.5 × 10^−9^, for the cosine similarity: 0, and for the total mRNA KDE: 2.4 × 10^−7^) while utilizing similar computational resources (Figure 5I,J).

## 3 Discussion

The most recent wave of high‐resolution full‐transcriptome in situ captures SRT methods is an attractive choice for characterizing cellular heterogeneity in tissue sections, however, to date there is a lack of performant methods to analyze these types of data. Furthermore, as there is no “one size fits all” workflow, analysis needs to be exploratory and interactive, e.g., to optimize the clustering parameters to obtain the correct number of clusters or to test the efficacy of annotating spatial gene expression from signature sets obtained from different single‐cell studies. Such an interactive analysis paradigm demands fast and efficient algorithms with minimal computational requirements.

In this study, we present a novel computational tool, Sainsc. Applying Sainsc to simulated data, we achieved top‐tier classification accuracy and demonstrated that our cell‐type assignment score can be used as a filter to improve the confidence of the results. We then accurately mapped the cellular landscape at the whole‐organism level, improving on previous segmentation‐based analysis. We demonstrate that Sainsc was the most computationally performant when analysing the mouse brain requiring only ≈1 hour of CPU wall time and ≈5 GB of memory. The moderate resource requirements of our implementation will facilitate exploratory analysis of extremely large datasets. Furthermore, Sainsc's annotations can easily be scaled up to several hundred cell types by integrating the expression landscape of thousands of genes.

We demonstrate the additional utility of the assignment score for interpreting the quality of the results. While annotating cell types based on gene expression signatures obtained from single‐cell RNAseq is a quick way to contextualize new data in the light of prior knowledge, we show that when the gene panels or cell type definitions differ from the reference source, it can result in lower confidence cell‐type assignment. To the best of our knowledge, we are the first to demonstrate the value of a visual map of the assignment score to effectively evaluate and improve assignment confidence at the organ and organism scales.

We also compared Sainsc to two other existing tools in this space, TopACT and FICTURE. While TopACT was highly accurate, its computational inefficiency makes it unsuitable for exploratory analysis. We were unable to complete the analysis of the mouse coronal hemibrain Stereo‐seq sample with TopACT in under seven days. FICTURE performed similarly to Sainsc on the same gene set, however, in our analysis we noticed that if chosen appropriately, much fewer genes are necessary to distinguish cell types accurately (e.g., for the Xenium dataset 248 genes are sufficient to separate 47 cell types). This reduced number of genes comes with great performance benefits for Sainsc, especially in terms of runtime. Taken together, we believe that Sainsc is currently the only computational tool capable of accurately analysing such large datasets with resource requirements that approach the scope of consumer electronics. Further emphasis is placed on efficiency when considering the spatial genomics field is going: toward 3D cell atlas,^[^ ^43^ ^]^ multi‐omics,^[^ ^44^ ^]^ and entire human organs.^[^ ^45^ ^]^ We believe Sainsc is uniquely suited to be further developed to meet the even higher computational needs of increasingly complex experiments without sacrificing accuracy.

## 4 Experimental Section

All analyses were performed in Python (v3.10.14) with Sainsc (v0.0.1, compiled using rustc v1.76.0, unless otherwise specified) and the following main packages; pandas (v2.2.2), polars (v0.20.31), scipy (v1.14.0), numpy (v1.26.4), scanpy (v1.10.2), squidpy (v1.5.0), anndata (v0.10.8).

### Sainsc—Gene Expression Estimation

Sainsc adopts the estimation of gene expression in 2D based on kernel density estimation from SSAM.^[^ ^19^ ^]^ In short, the gene expression density $\hat{p}$ is estimated as

(1) $$\hat{p}\left(x\right)=\frac{1}{N}\;\sum_{i}^{N}\kappa_{h}\left(x-x_{i}\right)$$

where κ_*h*_ is the kernel function in dependence of the bandwidth *h*, *N* the number of data points, and *x_i_* the location of the *i*th spot of the given gene. As a simplification, however, a discrete space (pixels) was only considered for both the gene expression and the estimated density, which allows for precomputed kernel “masks” and efficient calculation.

### Sainsc—Cell‐Type Assignment

Cell‐type assignment to each spot (pixel) is based on cosine similarity. The cosine similarity measures the similarity of two vectors via their angle and can be efficiently computed using the inner product.

(2) $$S_{c}\left(\vec{x},\vec{y}\right)=\cos\left(\theta_{xy}\right)=\frac{\vec{x}\cdot \vec{y}}{∥\vec{x}{∥}_{2}∥\vec{y}{∥}_{2}}$$

The cosine similarity is calculated between a pixel and each cell‐type signature, and the most similar cell type is assigned.

### Sainsc—Assignment Score

Let $\vec{p}$ be the gene expression of a pixel (for all genes under consideration) and $\vec{a}$ / $\vec{b}$ the signatures of the most and 2nd most similar cell types, respectively. The assignment score is defined as

(3) $$AS=\frac{S_{c}\left(\vec{p},\vec{a}\right)-S_{c}\left(\vec{p},\vec{b}\right)}{\max_{x}S_{c}\left(\vec{x},\vec{a}\right)-S_{c}\left(\vec{x},\vec{b}\right)}$$

where $\max_{x}S_{c}\left(\vec{x},\vec{a}\right)-S_{c}\left(\vec{x},\vec{b}\right)$ is the upper bound of the difference in cosine similarities given $\vec{a}$ and $\vec{b}$. Assuming non‐negative gene expression the following must hold 0° ≤  θ_*pa*_ ≤  θ_*pb*_ ≤ 90°. For simplicity, we assume normalized cell‐type signatures, i.e., $∥\vec{a}{∥}_{2}=1$ and $∥\vec{b}{∥}_{2}=1$.

(4) $$\begin{array}{ccc} S_{c}\left(\vec{x},\vec{a}\right)-S_{c}\;\left(\vec{x},\vec{b}\right) & = & \cos\left(\theta_{xa}\right)\;-\;\cos\left(\theta_{xb}\right)=\frac{\vec{x}\cdot \vec{a}}{∥\vec{a}{∥}_{2}∥\vec{x}{∥}_{2}}\;-\;\frac{\vec{x}\cdot \vec{b}}{∥\vec{b}{∥}_{2}∥\vec{x}{∥}_{2}}\\ & = & \frac{\sum_{i}a_{i}x_{i}}{∥\vec{x}{∥}_{2}}\;-\;\frac{\sum_{i}b_{i}x_{i}}{∥\vec{x}{∥}_{2}}=\frac{\sum_{i}a_{i}x_{i}-b_{i}x_{i}}{∥\vec{x}{∥}_{2}}\\ & = & \frac{\sum_{i}x_{i}\left(a_{i}-b_{i}\right)}{∥\vec{x}{∥}_{2}}=\frac{\vec{x}}{∥\vec{x}{∥}_{2}}\;\cdot \left(\vec{a}-\vec{b}\right) \end{array}$$

The inner product of $\vec{x}$ and $\left(\vec{a}-\vec{b}\right)$ is maximal for colinear vectors and therefore $x_{i}=\{\begin{array}{c} a_{i}-b_{i},\;a_{i}-b_{i}>0\\ 0,\;otherwise \end{array}$ (where negative dimensions are set to 0 due to the non‐negativity constraint).

### Sainsc.jl

Conceptually, the Julia implementation was equivalent to the Python/Rust implementation, and most data structures (e.g., hashmap of same‐sized sparse matrices for counts) and processing steps (e.g., processing the data in chunks when generating the cell‐type map) were “identical.” As the ecosystem was still young and evolving, Sainsc.jl integrates with multiple single cell/spatial transcriptomics frameworks, including Muon.jl which allows writing the gene expression of local maxima to h5ad files, which can then be easily read from Python.

To compare the Julia with the Python/Rust implementation of Sainsc we analyzed the Stereo‐seq hemibrain dataset in Julia (v1.10.4) with Sainsc.jl (commit ea4cfdf031d937da783cf55351a33d693d4a5618) using the same parameters as described with the segmentation‐based cell‐type signatures. Results were plotted in Python to make them visually comparable.

### Synthetic Data

To compare Sainsc to TopACT the simulated was used data from the TopACT publication.^[^ ^27^ ^]^ Briefly, 625 cell centers were sampled from a uniform distribution and placed within a 500 × 500 grid and the cell outlines were defined by their Voronoi diagram. Each cell was at random assigned a cell type based on the corresponding cell type abundances. The gene expression for each spot was imputed based on a Poisson process and the corresponding cell type average gene expression profile. The 20% of spots were randomly chosen and their gene expression was set to zero to model the zero inflation. For Sainsc the bandwidth was set to five for the Gaussian kernel (truncated at 2× bandwidth) and to ten for the Epanechnikov kernel. The average cell‐type expression was used as cell‐type signatures for Sainsc and the accuracy was calculated using *sklearn.metrics.accuracy_score*. Results for TopACT and RCTD were taken from the publication.

### Stereo‐Seq Analysis

All Stereo‐seq datasets were analyzed at native resolution with 500 nm/px (equivalent to the 500 nm bead‐to‐bead distance). A Gaussian kernel with bandwidth 8 px (4 µm) truncated at two bandwidths was used throughout.

### Stereo‐Seq Analysis—Embryo

The E16.5 E1S3 embryo data from the original Stereo‐seq publication was cropped and masked to the region of interest. The cell‐type assignment was performed and for visualization, the background was filtered based on cell‐type‐specific and global thresholds for the KDE of the total mRNA. For the supervised analysis signatures were extracted from the segmented data provided in the original publication by selecting the top 2000 highly variable genes (HVG) (*scanpy.pp.highly_variable* with flavor “seurat_v3”) and averaged across all cells per cell type.

### Stereo‐Seq Analysis—Embryo Unsupervised

For the de novo analysis the data was read as 50 × 50 bins to identify spatially‐variable genes. The bins (“cells”) were filtered to contain more than 1000 counts and genes with less than 100 counts were excluded. The top 10000 HVG were identified using *scanpy.pp.highly_variable* with flavor “seurat_v3”. A spatial neighbor graph was built using *squidpy.gr.spatial_neighbors* (coord_type = “grid”, n_rings = 1, n_neighs = 4) and the top 2000 spatially‐variable genes by Moran's I of the 10000 HVGs were identified using *squidpy.gr.spatial_autocorr* (with mode = “moran”). Next, the data was loaded at native resolution (500 nm), and the local maxima identified (s*ainsc.LazyKDE.find_local_maxima* with min_dist = 4 and min_area = 60). The local maxima were loaded as AnnData objects using the previously identified SVGs, the counts per “cell” normalized (*scanpy.pp.normalize_total*) and log‐transformed (scanpy.pp.log1p). The data was PCA transformed (*scanpy.pp.pca*) and the neighborhood graph, based on the latent dimensions, was constructed (*scanpy.pp.neighbors*). The data was then clustered using Leiden community detection (*scanpy.tl.leiden* with resolution = 2). The “cell‐type” signatures were created as average per cluster and used for cell‐type assignment.

### Stereo‐Seq Analysis—Coronal Mouse Hemibrain

To compare Sainsc to the segmentation‐based cell type assignment of the mouse coronal hemibrain section, signatures were extracted as average per cell type for the differentially expressed genes (7972) reported in Yao et al.^[^ ^34^ ^]^ To demonstrate the efficiency in identifying the isocortex layers cell‐type signatures from scRNA‐seq data from Yao et al.^[^ ^33^ ^]^ were calculated per “subclass” (downsampled to 5000 cells per subclass) for the differentially expressed genes reported in the same study.

### Stereo‐Seq Analysis—Border‐Associated Macrophage Detection

To detect border‐associated macrophages (BAM), we generated cell‐type signatures from the Allen brain atlas (10× Genomics Chromium v2 data) from Yao et al.^[^ ^34^ ^]^ for all subclasses (downsampled to max. 500 cells per subclass) resulting in 279 signatures. Using the differentially expressed genes from the same publication the Stereo‐seq brain was annotated as previously described. The binary map of BAM assigned spots was labeled as connected regions using *skimage.measure.label* and the centroids extracted using *skimage.measure.regionprops_table* and filtered for regions with an area of at least 60 pixels.

### Xenium Analysis

The Xenium brain dataset from 10X genomics (replicate 1) was analyzed by binning all transcript counts to 500 nm. The cell‐type signatures were based on the segmented Xenium dataset by Salas et al.^[^ ^20^ ^]^ A Gaussian kernel with a bandwidth 8 px (4 µm) truncated at two bandwidths was used. To correctly assign cell types in the PVT region we used cell‐type signatures generated from downsampled scRNAseq data^[^ ^34^ ^]^ based on the “class”‐level. Additionally, the “PVT‐PT Ntrk1 Glut” subclass was added to the signatures, and the analysis repeated as described above.

### Nova‐ST Analysis

NovaST data was binned to 500 nm and analyzed using the signatures based on the Stereo‐seq segmented mouse hemibrain slice for the differentially expressed genes defined in Yao et al.^[^ ^34^ ^]^ with a Gaussian kernel of bandwidth, 8 px truncated at 2 bandwidths.

### Comparison to SSAM

To compare Sainsc to SSAM (v1.0.1) we analysed the Xenium brain dataset. The pixel size was set to 1 µm and the bandwidths to 2.5 µm (both SSAM defaults). The truncation of the Gaussian kernel was set to 4×bandwidths for sainsc (fixed parameter for SSAM). The cell types were classified using the signatures obtained from the segmented Xenium data (see above). SSAM was run in both, default and “fast KDE” mode. To evaluate the concordance of the cell type assignment the adjusted Rand index (*sklearn.metrics. adjusted_rand_score*) and the normalized mutual information was calculated (*sklearn.metrics.normalized_mutual_info_score*) for all foreground pixels (total mRNA KDE >0.1). The runtime comparison was done as described below while using 512 GB memory for SSAM and 16 GB memory for Sainsc.

### Benchmarking and Runtime Performance Metrics

The Stereo‐seq mouse brain sample was used to benchmark the scalability of Sainsc (v0.1.0 with polar v1.2.0) and compare the runtime performance against other methods. To calculate runtime performance metrics jobs were submitted using slurm *sbatch* to a single compute node exclusively (‐N 1 –exclusive) with 8 CPUs (‐n 8, unless otherwise specified) with each node consisting of a Dell PowerEdge C6420 with 2x Intel Xeon Gold 6252@ 2.1 GHz. Jobs were assigned 64 GB of RAM, unless otherwise specified. The metrics were calculated from the slurm job information (using “ElapsedRaw” as wall time, “TotalCPU” as CPU time, and “MaxRSS” as maximum memory usage) for the entire workflow including data loading, pre‐processing, and “spot”‐wise cell‐type/factor assignment.

### Benchmark—Sainsc

Sainsc was run with eight threads, and a Gaussian kernel of bandwidth eight truncated at two bandwidths. To evaluate scalability in terms of analyzed tissue area and number of transcripts, the tissue was cropped to the specified fraction of the area (cropping equally along both axis) and the transcripts were subsampled on a per‐gene basis. To compare the scalability with the number of genes and cell types, cell‐type signatures from Yao et al.^[^ ^33^ ^]^ and genes appearing in both the data and the signatures were randomly subsampled. Jobs for the scalability analysis were submitted with 32 GB of RAM. For the comparison with FICTURE and TopACT cell‐type assignment was performed for the 42 “subclasses” defined in Yao et al.^[^ ^33^ ^]^ and the number of genes for the signatures was varied; 7972 (differentially expressed genes from Yao et al. that are detected in the data), and the 19708 genes as used by FICTURE after its default filtering. For the multi‐threading comparison jobs were submitted with the indicated number of threads.

### Benchmark—FICTURE

For FICTURE (v0.0.3.1)^[^ ^32^ ^]^ the Makefile was generated using the *run_together* command and non‐essential processing steps were removed (e.g., plotting and bulk differential gene expression) resulting in an analysis consisting of the main steps *make_spatial_minibatch, make_dge, fit_model, transform*, and *slda_decode*. Furthermore, the *sort* commands were adjusted to a buffer size of 4 GB (‐S 4G). The analysis was performed with 42 factors (corresponding to the 42 cell types used for the other tools) and a width of 15 (corresponding to 8.7 µm side‐to‐side width of the hexagon following the original publication) while keeping other parameters at the default. The *make* command was run with 1 job in the case of single‐threaded FICTURE and with 2 jobs otherwise (‐j 1 and ‐j 2 respectively).

### Benchmark—TopACT

TopACT (v1.1.0)^[^ ^27^ ^]^ was trained on single‐cell data from Yao et al.^[^ ^33^ ^]^ The data was subsampled to at most 1000 cells per cell type leaving ≈37000 cells prior to running the analysis. For classification all pixels within the convex hull around the gene expression were considered and the classification was run in parallel on eight processes for a radius from 3 to 9 (min_scale and max_scale parameters, respectively) following the original publication, and the job was submitted using 128 GB of memory.

## Conflict of Interest

The authors declare no conflict of interest.

## Author Contributions

N.I. conceived the study. N.M.‐B. implemented Sainsc, Sainsc.jl, and performed data analysis and interpretation. S.T. assisted with the interpretation of mouse brain results and provided critical comments and feedback. N.I. and N.M.‐B. wrote the manuscript. All authors commented on and critically revised the manuscript.

## Supporting information



Supporting Information

Supporting Information

## Data Availability

All data used in this study is publicly available. Stereo‐seq mouse brain and embryo data was downloaded from the original Stereo‐seq study (https://db.cngb.org/stomics/mosta/).^[^ ^14^ ^]^ Xenium mouse brain data was obtained from 10x Genomics (https://www.10xgenomics.com/datasets/fresh‐frozen‐mouse‐brain‐replicates‐1‐standard). The cell annotation based on segmentation of the Xenium dataset from Salas et al.^[^ ^20^ ^]^ is available from Zenodo (https://doi.org/10.5281/zenodo.13821309). Nova‐ST mouse brain data^[^ ^17^ ^]^ was downloaded from Gene Expression Omnibus (https://www.ncbi.nlm.nih.gov/geo/query/acc.cgi?acc=GSM8093729). The scRNAseq data for the isocortex and hippocampal formation from Yao et al.^[^ ^33^ ^]^ was downloaded from the NeMO Archive (https://assets.nemoarchive.org/dat‐jb2f34y) and for the whole mouse brain from Yao et al.^[^ ^34^ ^]^ (https://portal.brain‐map.org/atlases‐and‐data/bkp/abc‐atlas). The Sainsc Python package is available at https://github.com/HiDiHlabs/sainsc and the Julia implementation at https://github.com/niklasmueboe/Sainsc.jl. The analysis code to reproduce the results of this study is deposited at https://github.com/HiDiHlabs/sainsc‐study.
