# Supplementary material for: Sainsc: A Computational Tool for Segmentation‐Free Analysis of In Situ Capture Data
Source: Small Methods. 2024 Nov 12;9(5):2401123. doi: 10.1002/smtd.202401123 (PMC12103232; doi:10.1002/smtd.202401123)
Supplement: Supplementary file 1 — Supporting Information [file SMTD-9-2401123-s001.docx]

# Supplementary Information

## Supplementary Figures


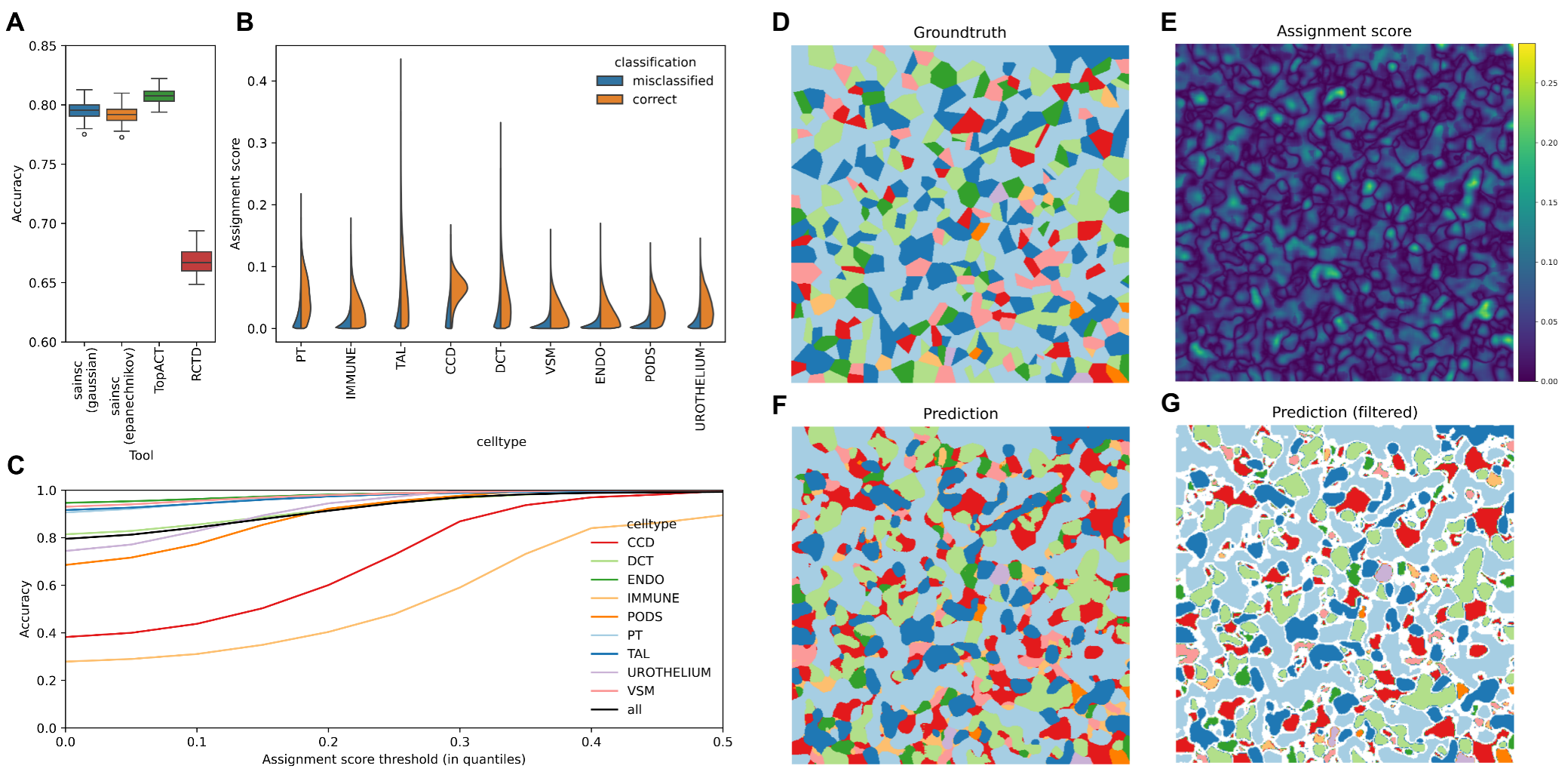


**Figure S1.** Sainsc cell-type assignments are accurate and increasing assignment score associates with increasing accuracy. (A) Box plot of accuracy of cell-type assignments in 100 simulated samples. From left to right: Sainsc using a Gaussian kernel (bandwidth of 5, truncated at 2 bandwidths), Sainsc with an Epanechnikov kernel (bandwidth of 10), TopACT, and RCTD. Center line, median; box limits, upper and lower quartiles; whiskers, 1.5x interquartile range; points, outliers. TopACT and RCTD results are taken from Benjamin *et al.* 2024. (B) Violin plots of cell-type-specific assignment confidence scores in correct and misclassified spots for Sainsc with Gaussian kernel. Misclassified spots show very low assignment scores compared to correctly assigned spots. (C) Line plot of change in Sainsc accuracy when filtering the bottom percentiles of assignment scores. Visualisation of one of the replicates of cell-type map for (D) the ground truth, (E) assignment score map, (F) Sainsc, (G) and Sainsc filtered for bottom 20^th^ percentile of assignments per cell type. CCD, cortical collecting duct; DCT, distal convoluted tubule; ECS, endothelial cells; PT, proximal tubule; TAL, thick ascending limb of the loop of Henle; VSM, vascular smooth muscle.


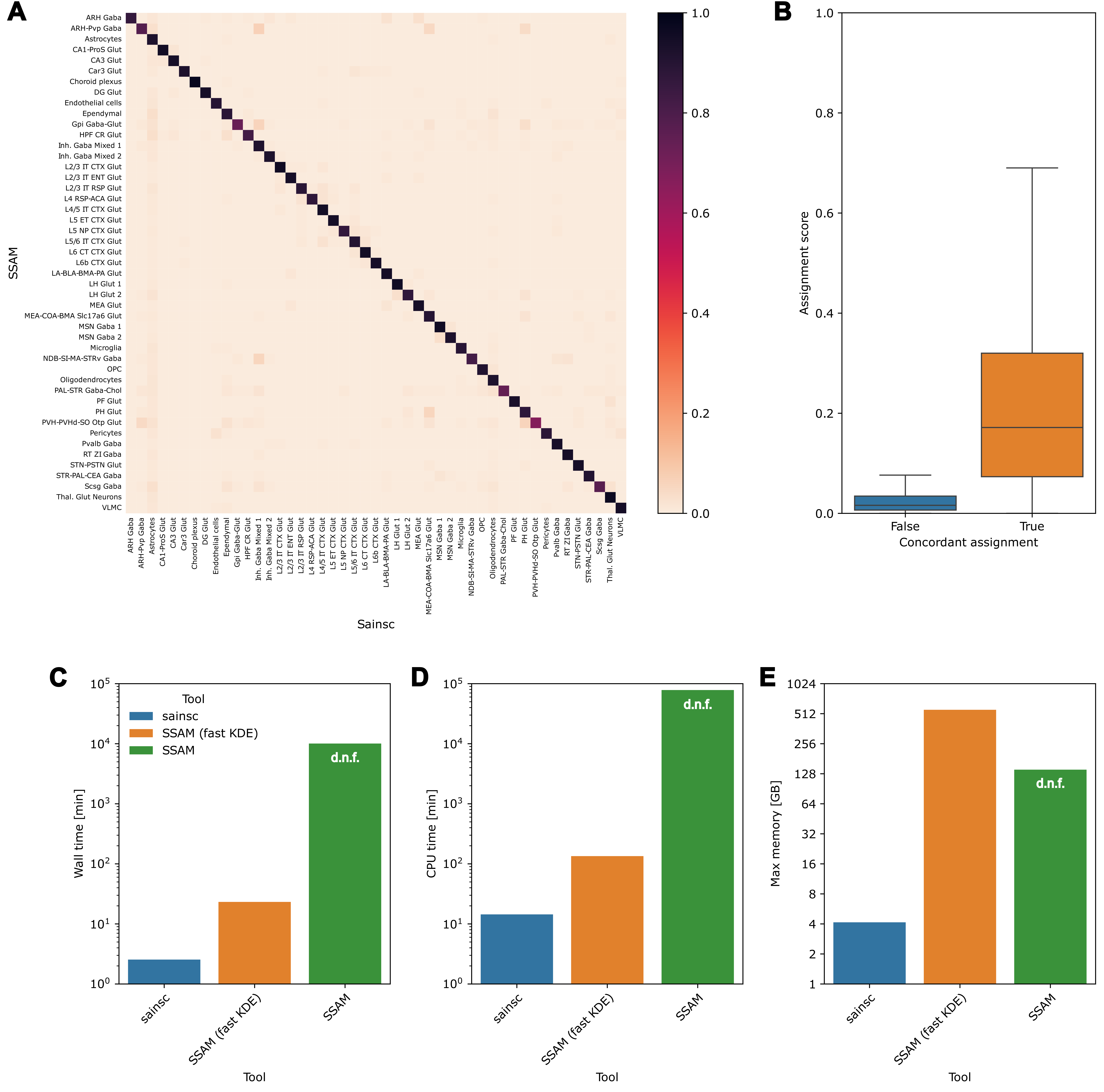


**Figure S2.** Sainsc has improved performance compared to SSAM. (A) Sainsc generates highly similar results to SSAM demonstrated by concordance in cell type assignment on the Stereo-seq mouse hemi-brain section. (B) Discordant pixels have a lower assignment score than concordant assignments (3,559,749 / 45,159,261 pixels for discordant and concordant pixel assignment of SSAM and Sainsc, respectively). Center line, median; box limits, upper and lower quartiles; whiskers, 1.5x interquartile range; outliers not shown. Sainsc outperforms SSAM by orders of magnitude for (C) wall time, (D) CPU time, and (E) maximum memory usage. d.n.f.; did not finish.


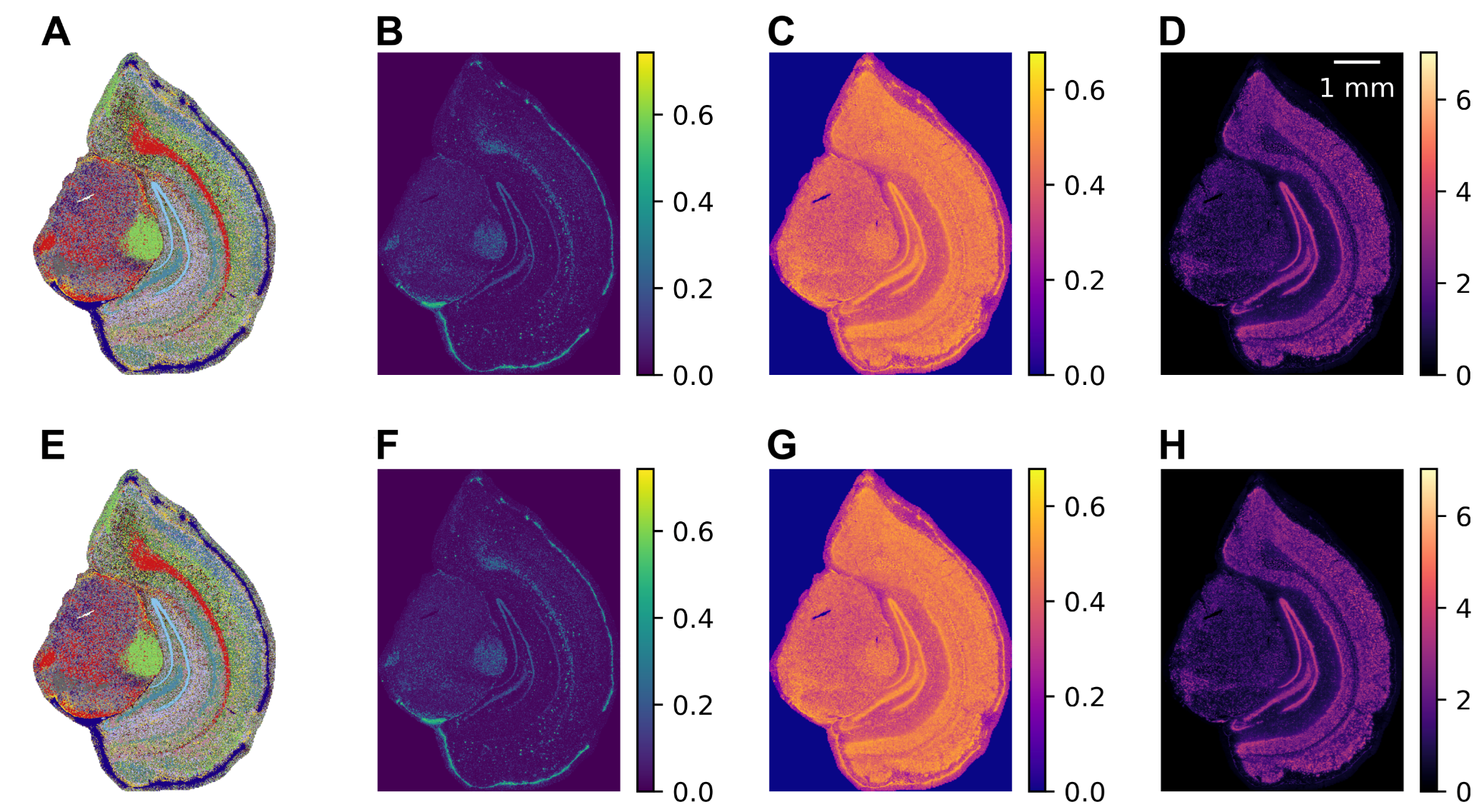


**Figure S3.** Sainsc’s Python and Julia implementation generate near identical results. Comparison of the Stereo-seq mouse hemibrain when analysed with (A-D) Sainsc (Python) and (E-H) Sainsc.jl (Julia). Shown are (A, E) the unfiltered cell-type map, (B, F) the assignment score, (C, G) the cosine similarity of the assigned cell type, and (D, H) the KDE of the total mRNA.
